# Obesity-associated asthma in childhood 

**DOI:** 10.5414/ALX02178E

**Published:** 2020-10-19

**Authors:** Maria Mangova, Tobias Lipek, Maike vom Hove, Antje Körner, Wieland Kiess, Regina Treudler, Freerk Prenzel

**Affiliations:** 1 University of Leipzig Medical Center, Department for Pediatrics,; 2 LICA – Leipzig Interdisciplinary Center for Allergy (Comprehensive Allergy Center), and; 3 University of Leipzig Medical Center, Department of Dermatology, Venereology and Allergy, Leipzig, Germany; * M. Mangova und T. Lipek contributed equally to this work.

**Keywords:** inflammation, lung function, comorbidities, quality of life, asthma therapy

## Abstract

Obesity and bronchial asthma are very common diseases in children and adolescents, associated with a considerable burden of disease, reduced quality of life and comorbidities. Obesity is a significant risk factor for bronchial asthma. On the one hand, obesity leads to changes in the mechanics and function of the lungs and chest. On the other hand, obesity-associated inflammatory processes with increased production of leptin and cytokines may trigger bronchial inflammation with the appearance of asthmatic symptoms. The diseases are also linked by genetic factors. Physical activity and weight reduction have a significant benefit. Pharmacotherapy must be based on the pattern of inflammation. This article summarizes the current state of the literature on the association of asthma and obesity and presents current and possible future treatment options.

## Introduction 

Asthma and obesity are two of the most common diseases in pediatrics, the prevalence of which has increased in parallel over the last decades and has recently reached a plateau [1, 2, 3]. Bronchial asthma is pathophysiologically defined as an inflammatory respiratory disease with symptoms that vary in time and intensity, such as shortness of breath, wheezing, chest tightness and coughing, and bronchial hyperresponsiveness. There is a complex interaction of endogenous and genetic factors and exogenous stimuli of physical, chemical, pharmacological, and immunological nature resulting in mucus hypersecretion, bronchospasm, mucosal edema, and remodeling [1]. 

Obese children often have dyspnea and other respiratory problems, but these are not always due to asthma. Overweight and obesity in children is a clear risk factor for bronchial asthma, especially in girls [1, 4]. When obese children develop asthma, they have a more severe disease phenotype associated with a higher burden of disease, reduced quality of life, greater frequency of exacerbations and hospitalization, and a suboptimal response to commonly used asthma medicines [5, 6, 7, 8]. The mechanisms underlying obesity-associated asthma are not yet fully understood. However, there is increasing evidence that asthma is associated with systemic inflammation of fatty tissue [7, 9, 10]. This paper provides an up-to-date literature review on the association of childhood obesity and asthma and presents current and possible future therapeutic strategies. 

## Units of measurement for obesity and influencing factors on bronchial asthma 

Obesity is present when the proportion of body fat in the total body mass is pathologically increased. Since the fat percentage of the body can only be determined exactly with elaborate methods, the use of the easily measurable parameters height and body weight as well as the body mass index (BMI = body weight/height^2^ (kg/m^2^)) derived from these parameters for estimating the body fat percentage in adults has become established worldwide. In accordance with the specifications of the European Childhood Obesity Group, the Working Group on Obesity in Childhood and Adolescence recommends the use of the 90^th^ or 97^th^ age- and gender-specific BMI percentile as a limit value for defining overweight or obesity. Extreme obesity is defined as a BMI > 99.5^th^ percentile [11]. 

Many prospective studies on obesity and asthma have primarily used the BMI as an indicator of obesity. Other indicators have been measures of fat distribution, such as skin-fold thickness, waist and hip circumference, and body composition [12, 13, 14]. 

The association of obesity and asthma is modified by many influencing factors. In a German cohort study, evidence was found that obesity is only independently associated with asthma among children with an atopic disposition [13]. Gender also seems to play a role: In a large prospective longitudinal study in children, Wadden et al. [15] showed an additional effect of the female sex on increasing BMI and persistent asthma. The potential anti-inflammatory hormone effect of testosterone and the pro-inflammatory effect of estrogen are discussed, as it has been shown that asthma occurs more frequently in early childhood in boys and postpubertal in girls [16]. 

## Relationship between obesity and lung function 

**Figure 1.**
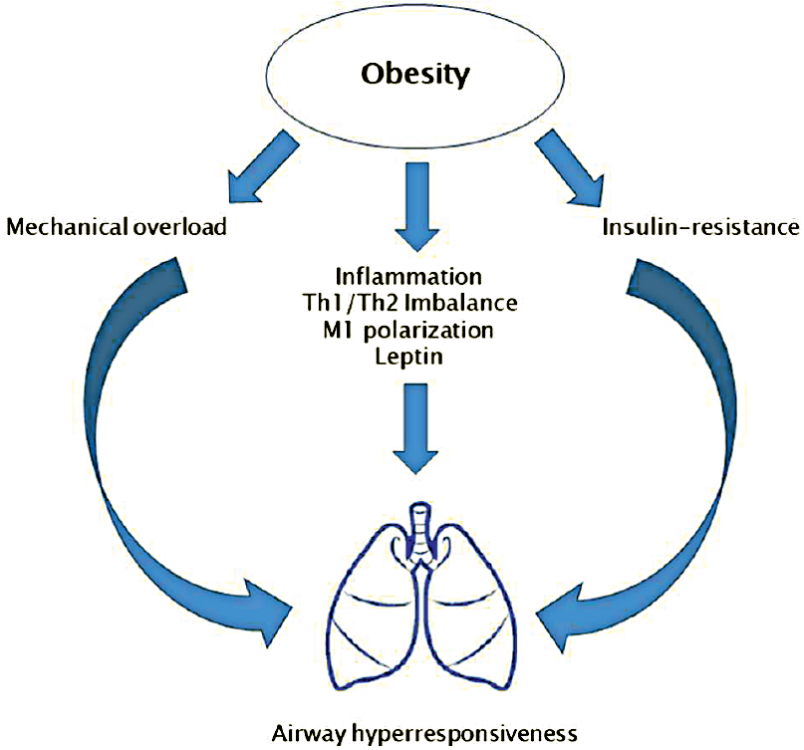
Possible pathomechanisms of obesity-associated asthma. Modified reprint under the open access conditions of the international Creative Commons Attribution License (CC BY 4.0) https://creativecommons.org/licenses/by/4.0/ from Umano, Giuseppina Rosaria; Pistone, Carmelo; Tondina, Enrico; Moiraghi, Alice; Lauretta, Daria; Miraglia Del Giudice, Emanuele; Brambilla, Ilaria (2019): Pediatric Obesity and the Immune System. In: Frontiers in pediatrics 7, p. 487. DOI: 10.3389/fped.2019.00487. Copyright ^©^ 2019 Umano, Pistone, Tondina, Moiraghi, Lauretta, Miraglia del Giudice and Brambilla.

Obesity causes significant changes in the mechanics of the lungs and thorax with reduction in pulmonary compliance. This alters the breathing pattern and probably contributes to respiratory symptoms such as wheezing, dyspnea, and orthopnea. The increased intra-abdominal and pleural pressures lead to a reduction in expiratory reserve volume (ERV) and functional residual capacity (FRC) (Figure 1) [8]. 

Studies have shown that children with obesity tend to have large lungs, but their airways are normally sized. This mismatch between lung tissue growth and airway caliber is called “airway dysanapsis” [17]. As a result, obese children have normal forced expiratory volume in one second (FEV1) and normal expiratory flow with increased forced vital capacity (FVC) compared to normal-weight children. Accordingly, the FEV1/FVC ratio (Tiffeneau index) used for the diagnosis of asthma is reduced [17, 18, 19, 20, 21, 22]. Since an important cause of the symptoms could therefore be mechanical rather than inflammatory, it is not surprising that a reduced response to inhaled corticosteroids is often observed in obese children with respiratory dysanapsis [18, 23]. 

In other studies, however, no clear correlation has been found between BMI and lung function parameters in children with asthma [24]. The concept of dysanapsis requires further prospective evaluation and does not appear to be sufficient on its own to describe the association between asthma and obesity. 

## Inflammation pattern in obesity-associated asthma 

**Figure 2.**
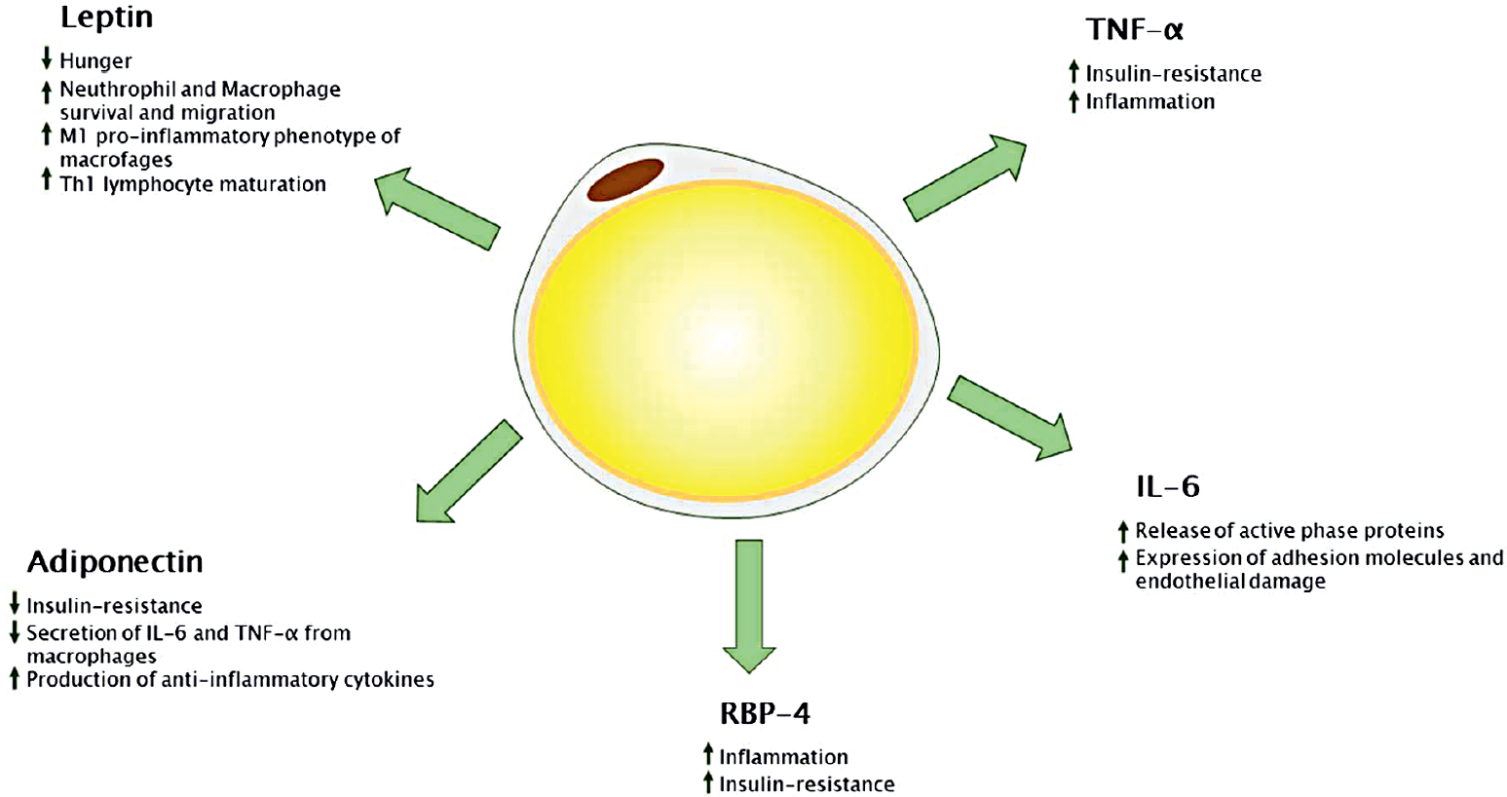
Adipokines and their immunological effect. Modified reprint under the open access conditions of the international Creative Commons Attribution License (CC BY 4.0) https://creativecommons.org/licenses/by/4.0/ from Umano, Giuseppina Rosaria; Pistone, Carmelo; Tondina, Enrico; Moiraghi, Alice; Lauretta, Daria; Miraglia Del Giudice, Emanuele; Brambilla, Ilaria (2019): Pediatric Obesity and the Immune System. In: Frontiers in pediatrics 7, p. 487. DOI: 10.3389/fped.2019.00487. Copyright ^©^ 2019 Umano, Pistone, Tondina, Moiraghi, Lauretta, Miraglia del Giudice and Brambilla.

Fatty tissue has been in the focus of science for quite some time because of the many negative health effects of overweight. With regard to asthma, the pro-inflammatory effects of adipose tissue and the potential role of this inflammation as an effect modifier on bronchial asthma have been investigated in several studies (Figure 1, and Figure 2) [25, 26, 27, 28]. In normal-weight people, adipose tissue typically secretes small amounts of pro-inflammatory cytokines such as IL-6, IL-8, tumor necrosis factor-α (TNF-α) and adipokines such as leptin, and produces large amounts of the anti-inflammatory adipokine adiponectin. In the obese state, fatty tissue hypertrophies and is infiltrated by pro-inflammatory macrophages. These activated macrophages and hypertrophied adipocytes produce increased pro-inflammatory cytokines and leptin and decreased adiponectin. This metabolic inflammation is believed to cause the systemic complications of obesity such as type 2 diabetes, hepatic steatosis, and metabolic syndrome [9, 15, 29, 30]. 

This has led to the assumption that the same mechanism may be involved in bronchial inflammation in asthma. This is supported by the fact that systemic inﬂammation is more pronounced in patients with obesity-associated asthma than in obese patients without asthma [31]. Of central importance is the adipokine leptin, which plays an important role in embryonic and fetal development and in controlling body fat stores by regulating eating habits, metabolism, the autonomic nervous system, and the body’s energy balance [32]. However, leptin is also involved in the regulation of the respiratory drive, surfactant production, and lung development in newborns [33, 34, 35, 36]. Given these functions, leptin may well be involved in the pathogenesis of respiratory diseases. Levels of leptin are higher in obese children and adults than in non-obese ones [30, 37]. However, the expression of leptin is significantly increased in obese children and adults with asthma compared to obese patients without asthma [9, 26]. Children with uncontrolled asthma had higher leptin levels than those with controlled asthma (Figure 2) [26]. 

In addition, Zheng et al. [27] suggested that leptin acts as a key risk factor for the development of allergic asthma in obese individuals through the induction of the unfolded protein response factor XBP1s, which stimulates pro-allergic lymphocyte survival and cytokine production. This would build a bridge to the above-mentioned finding that obesity is a risk factor especially in connection with atopy [13]. 

The macrophages of fatty tissue are significantly elevated and mediate both local and systemic inflammation, leading to the recruitment and activation of type 1 T-helper cells [9, 38, 39, 40]. Their activation enhances the immune response by producing additional pro-inﬂammatory cytokines and inflammatory markers such as TNF-α, IL-8 and IL-6, highly sensitive C-reactive protein (hs-CRP) and monocytic chemoattractive protein-1 (MCP-1) [41, 42, 43, 44]. The latter causes an activation of monocytes and their transformation into macrophages [10]. 

The influence of this systemic inflammation on the bronchial system has not yet been fully clarified. However, it is known that TNF-α is elevated in uncontrolled, severe asthma [45]. The above-mentioned pro-inflammatory cytokines can influence lung function by activating vascular endothelial cells, respiratory fibroblasts, smooth muscle cells, or respiratory epithelial cells [46]. In addition, IL-6 affects the differentiation of naive T cells into Th17 cells and may indirectly affect lung function through the action of IL-17 [47, 48]. Furthermore, TNF-α is involved in the recruitment of neutrophils and eosinophils and the activation of T cells in the airways. Thus, there are connections between type 1 (TH2-low) and type 2 (TH2-high) inflammation [48, 49, 50]. This and the activation of congenital type 2 lymphoid cells (ILC2s) could explain why elevated T2 cytokines such as IL-4 and IL-5 are found in asthma in parallel to an increase in TNF-α [26, 44]. 

IL-4 stimulates B-cell and T-cell proliferation and the differentiation of CD4+ T cells into Th2 cells. IL-4 also plays a central role in the regulation of IgE synthesis and induces the expression of the low-affinity IgE receptor on macrophages. IL-5 is an activator for eosinophils and is therefore considered a central cytokine in allergen- and parasite-mediated eosinophilic reactions. IL-5 stimulates B-cell growth and increases immunoglobulin secretion [49]. Nevertheless, no increase in fractional exhaled nitric oxide (FENO) as an eosinophilic inflammation marker has been shown in obese children [51]. 

In the literature, obesity-associated asthma is described on the one hand as being associated with neutrophilic or paucigranulocytic airway inflammation, and thus T2-low [52]. On the other hand, increased IL-5 and submucosal eosinophilia have been found in the sputum of obese patients with severe asthma [53]. 

In another study, no significant difference was found histologically in lung biopsies of adults with mild to moderate asthma compared to a control group of obese patients without asthma [54]. 

It is possible that specific inflammatory mechanisms such as the CDC42 pathway also play a role in obesity asthma [10]. 

In summary, obesity-associated asthma is characterized by a systemic inflammation of adipose tissue with cytokines and markers that initially correspond to a non-eosinophilic T2-low inflammation. However, there appears to be an overlap with T2-high asthma, which may be clinically significant. It may also be necessary to distinguish between obesity-exacerbated asthma and obesity-induced asthma [52]. 

Further elucidation of these pathogenetic mechanisms is of great importance for the development of targeted therapeutic strategies. 

## Genetics and epigenetics of obesity and asthma 

The different efficacy of steroids in the treatment of asthma in children with and without obesity is a clinically significant finding, which led to the investigation of molecular-genetic causes. At birth it is not yet completely decided whether a child becomes overweight. Therefore, postnatal epigenetic changes in particular are considered. Using single nucleotide polymorphisms (SNP), it can be said that the epigenetic footprint of immune cells of obese children with bronchial asthma differs from that of obese children without asthma and normal-weight children with asthma [31]. Granell et al. [55] confirmed a positive association between BMI and asthma in middle childhood in their population-based birth cohort study and found strong evidence that this association is due to a causal effect of BMI on asthma using randomization analyses based on 32 BMI-associated SNPs. 

Some genes have been identified as associated with asthma and obesity [56]. This includes on chromosome 5q31-32 the genes *ADRB2*, which encodes the adrenergic β2 receptor, and *NR3C1*, which codes for the glucocorticoid receptor. Interestingly, these are both genes that are important in the context of therapy. Furthermore, on chromosome 6p21.3 this concerns the TNF-α gene complex and on chromosome 11q13 the genes *UCP2, UCP3,* and the low-affinity immunoglobulin E-receptor gene. 

Future studies will hopefully provide further insights into the genetic influence of the association between obesity and asthma. 

## Comorbidities and quality of life in children with asthma and obesity 

Asthma is associated with metabolic dysregulation including insulin resistance, dyslipidemia, and metabolic syndrome. The prevalence of metabolic dysregulation such as insulin resistance and dyslipidemia, especially low levels of high-density lipoprotein (HDL), is higher in obese children with asthma than in normal-weight children with asthma [57, 58, 59]. Asthma and obesity also have some overlapping comorbidities such as psychosocial problems including depression and anxiety disorders, upper respiratory tract disorders, obstructive sleep apnea syndrome, dysfunctional respiratory symptoms, and gastroesophageal reflux [1, 60, 61, 62]. 

These overlapping comorbidities could be mutually reinforcing and must therefore be included in therapy. On the other hand, some comorbidities of obesity, such as obstructive sleep apnea and gastroesophageal reflux, may simulate asthma symptoms and lead to misinterpretation and the use of unnecessary anti-asthmatic medication that does not result in clinical improvement. 

Many studies have concluded that the quality of life in children with asthma and obesity is significantly lower in the long term than in healthy subjects. Physical activity was the most severely restricted [24]. 

## Treatment 

### Sport, diet, and socio-economic factors 

Today’s sedentary lifestyle, with the use of social media, electronic devices, television, and video games, has reduced active time outdoors and is therefore associated with overweight and obesity in children [63]. If obesity is one of the causes of changes in lung mechanics and function, weight loss should be able to reverse these changes. Aerobic fitness and physical activity clearly play a role in treating childhood obesity and asthma [7, 64]. The number of studies on the effect of weight loss on childhood asthma associated with obesity is limited. However, a benefit has been shown in terms of lung function, asthma control and symptoms, the severity of exercise-induced bronchoconstriction, and quality of life [65, 66, 67, 68, 69, 70]. 

In adult patients who have undergone bariatric surgery, an improvement in airway hyperreactivity, in lung volume and function of the small airways as well as in the systemic inflammatory situation have been observed [71, 72, 73]. 

### Pharmacotherapy 

Children with obesity and asthma should be treated according to current guidelines, which include therapy with inhaled corticosteroids (ICS), especially if there is evidence of allergic disease [1, 7]. It should be noted, however, that a reduced response to medication and poorer disease control is observed in this patient group [74, 75, 76, 77, 78]. 

While a number of biologicals are already available for T2-high asthma [79, 80], the therapeutic options for obesity-associated asthma are unclear. In this phenotype, as outlined above, the pattern of inflammation has not yet been sufficiently characterized. TNF-α receptor blockers initially showed encouraging results, but these could not be confirmed [45, 81]. Therapy with an anti-TNF-α antibody did not show a favorable adverse event profile, and anti-IL-17 therapy did not result in an improvement [52, 82]. The macrolide azithromycin could reduce the rate of exacerbation in adult patients with non-eosinophilic asthma [52, 80]. Interestingly, a reduction in BMI was observed in asthma under anti-IL-5 therapy, which was associated with the role of eosinophils in the homeostasis of these cells in adipose tissue [83]. 

### Future therapeutic options 

Since insulin resistance and a metabolic syndrome lead to a worsening of bronchial asthma and lung function, therapy of these complications is also useful with regard to pulmonary disease [84, 85]. Initial data in adults have shown that metformin may also improve asthma exacerbations in patients with metabolic syndrome [86]. The role of anti-IL-5 therapy needs further evaluation [53, 83], and an anti-IL-1 antibody is still being investigated in clinical trials [52]. Anti-thymic stromal lymphopoietin (TSLP) therapy, for example with tezepelumab, is also a possible approach [87]. 

Liraglutide can be used in the treatment of diabetes mellitus type 2 and severe obesity. In addition, improvements in bronchial asthma have been described in a mouse model [88]. 

## Conclusion 

The prevalence of asthma and obesity in children and adolescents has increased in many countries. Obese children have a higher risk of developing asthma than normal-weight children [89]. Obesity-associated asthma is associated with a higher burden of disease and associated reduced quality of life, reduced lung function and disease control, a higher incidence of exacerbations and hospitalization, and a suboptimal response to commonly used asthma medication [5, 6, 90]. As obesity can cause respiratory symptoms, there is a risk of overdiagnosis [1]. 

Obesity-associated asthma probably develops from a systemic inflammation originating in fatty tissue that is associated with an imbalance of leptin and adiponectin. Phenotypically, T2-low inflammation seems to be predominant, but overlaps with T2-high asthma are observed. 

Asthma should always be diagnostically confirmed, whereas FEV1 and expiratory flow restriction should rather be used for the detection of pulmonary obstruction due to respiratory dysanapsis. Evidence of bronchial hyperresponsiveness may confirm asthma; a low FENO indicates T2-high asthma. 

Also on the genetic level and in the case of other comorbidities, associations between obesity and asthma arise that may be therapeutically relevant. 

Therapeutically, weight reduction and sports are important interventions with clear clinical benefits. Pharmacotherapy is performed according to guidelines including ICS, whereby a benefit must be verified and an uncritical use of high-dose ICS must be avoided. 

Future research should clarify the pathomechanisms of obesity-associated asthma and help to better characterize this phenotype and sub-phenotypes. Based on this, new therapeutic approaches must be identified and reviewed. Primary prevention strategies and ways to implement lifestyle interventions must be developed. 

## Essential sentence 

Obesity and asthma in children are associated by inflammatory and mechanical processes that must be taken into account in therapy. 

## Funding 

No external funding. 

## Conflict of interest 

The authors declare that there is no conflict of interest.
